# Diagnostic performance of an automated plasma p-tau217 chemiluminescent assay for detecting Aβ pathology in a Chinese memory clinic cohort

**DOI:** 10.1016/j.tjpad.2026.100613

**Published:** 2026-06-05

**Authors:** Shuai Chen, Feng-Yu Wang, Rong Li, Chang Fu, Jing-Yu Shao, Yu Shen, Kai Ma, Xiao-Di Hao, Lin Cao, Jun-Ling Xu, Jie-Wen Zhang

**Affiliations:** a Department of Neurology, Zhengzhou University People's Hospital (Henan Provincial People's Hospital), Zhengzhou 450003, China; b Department of Nuclear Medicine, Zhengzhou University People's Hospital (Henan Provincial People's Hospital), Zhengzhou 450003, China; c Department of Medical Imaging, Zhengzhou University People's Hospital (Henan Provincial People's Hospital), Zhengzhou 450003, China; d Vazyme Biotech Co., Ltd, Nanjing 210046, China

**Keywords:** Alzheimer's disease, Plasma biomarker, Aβ pathology, Diagnostic performance

## Abstract

**Background:**

Blood-based biomarkers have emerged as promising tools for detecting Alzheimer’s disease (AD) pathology, but validation of automated plasma assays in Chinese clinical populations remains limited. This study evaluated the diagnostic performance of a fully automated chemiluminescent plasma biomarker assay for detecting amyloid-β (Aβ) pathology in a Chinese memory clinic cohort under different pre-analytical conditions.

**Methods:**

We enrolled 409 cognitively impaired participants from a single-center memory clinic, using amyloid-β positron emission tomography (Aβ-PET) as the reference standard. Plasma samples were analyzed under two pre-analytical conditions: frozen batch-processed samples from a historical cohort (n = 198) and freshly collected samples analyzed in real time in a prospective cohort (n = 211). Additionally, 95 participants underwent tau-PET imaging. Six plasma biomarkers were quantified using the Vazyme® AD Assay.

**Results:**

Across cohorts, p-tau217, p-tau217/Aβ42 ratio, and NfL/p-tau217 ratio consistently achieved excellent diagnostic performance (AUCs 0.92–0.95), followed by p-tau181 (AUCs 0.86–0.90). GFAP (AUCs 0.82–0.83) and the Aβ42/40 ratio (AUCs 0.76–0.81) showed moderate discriminative performance. Plasma p-tau217 alone achieved diagnostic accuracy comparable to composite biomarker models. A dual cut-point strategy reduced the indeterminate zone to <30%, with positive predictive values of 0.97–0.99 and negative predictive values of 0.86–0.87. Plasma p-tau217 was also significantly associated with tau-PET burden in both meta-temporal and neocortical regions (P < 0.001).

**Conclusion:**

This automated chemiluminescent plasma biomarker assay demonstrated high diagnostic accuracy for detecting Aβ pathology in a Chinese memory clinic cohort under different pre-analytical conditions. The findings support its potential utility as a practical blood-based biomarker approach in specialized clinical settings, while further multicenter studies are needed to confirm its generalizability across broader populations and healthcare environments.

**Abbreviations**
AD: Alzheimer's disease
PET: positron emission tomography
Aβ: amyloid-β
CDR: dementia rating scale
ADL: activities of daily living scale
H-AVLT: Huashan auditory verbal learning test
VFT: verbal fluency test
BNT: Boston naming test
MCI: mild cognitive impairment
MRI: magnetic resonance imaging
SUVRs: standardized uptake value ratios
ROI: region of Interest
GFAP: glial fibrillary acidic protein
NFL: neurofilament light chain
APOE: apolipoprotein E
SNP: single-nucleotide polymorphism
IQR: interquartile ranges
ROC: receiver operating characteristic
AUC: area under the curve
PPV: positive predictive value
NPV: negative predictive value

## 1 Background

Alzheimer's disease (AD) is the most common neurodegenerative disorder worldwide. In China, it presents with distinctive characteristics, including a large patient population, rapidly increasing prevalence, and a substantial socioeconomic burden. Epidemiological surveys estimate that approximately 15 million Chinese individuals aged ≥ 60 years have dementia, including 9.8 million with AD, accounting for approximately 25% of all AD cases worldwide [1]. As a developing country, China faces additional challenges, including low educational levels among the elderly population, limited public awareness, and an uneven distribution of healthcare resources. These factors collectively impede the timely and precise diagnosis of AD. Studies have reported clinical underdiagnosis rates of up to 76.8% in China, with an average diagnostic delay exceeding two years [2]. In the era of anti-amyloid monoclonal antibody therapies, such delays prevent patients from receiving timely disease-modifying interventions, thereby exacerbating economic and caregiving burdens on families and society. Therefore, developing efficient, readily applicable, and cost-effective biomarker assays is essential to address the dilemma of "high prevalence but inadequate diagnosis".

Advances in ultrasensitive detection technologies have established plasma biomarkers as a promising alternative to traditional approaches, such as positron emission tomography (PET) and cerebrospinal fluid analysis, for identifying AD-related pathologies, including amyloid-β deposition, tau pathology, neurodegeneration, and neuroinflammation. Plasma biomarkers, particularly p-tau217, have been extensively validated in Western populations for predicting Aβ pathology and clinical progression [3,4]. In a recent meta-analysis of 29,625 individuals, plasma p-tau217 showed the highest diagnostic accuracy for biologically defined AD, with a pooled sensitivity of 88.1%, specificity of 88.7%, and an AUC of 91.1% [5]. However, validation studies in Chinese cohorts remain scarce. Limited validation studies in China, primarily employing the single-molecule arrays (Simoa) and Lumipulse G platforms, have demonstrated comparable predictive performance to studies abroad [[6], [7], [8], [9], [10]].

The clinical translation of plasma biomarker assays in China remains at an early stage, and plasma biomarker testing may face practical challenges related to differences in laboratory infrastructure, workflow standardization, and sample processing conditions across healthcare settings. In 2024, the National Medical Products Administration (NMPA) of China approved a novel plasma-based AD biomarker assay implemented on a fully automated chemiluminescent immunoassay platform. Therefore, validation of newly developed p-tau217 antibody configurations across different assay architectures and real-world clinical settings remains important for assessing the reproducibility, robustness, and potential clinical applicability of this biomarker in Chinese populations.

In the present study, we evaluated the diagnostic performance of this automated chemiluminescent plasma biomarker assay for detecting Aβ pathology in a tertiary memory clinic cohort using Aβ-PET as the reference standard. To assess the robustness of the assay under different pre-analytical conditions, we compared biomarker performance between historical frozen plasma samples and freshly collected plasma samples analyzed in real time. We additionally evaluated the diagnostic performance of multiple plasma biomarkers and biomarker combinations and explored the potential influence of clinical and pre-analytical variables on plasma p-tau217 concentrations.

## 2 Methods

### 2.1 Participants

**Fig. 1:**
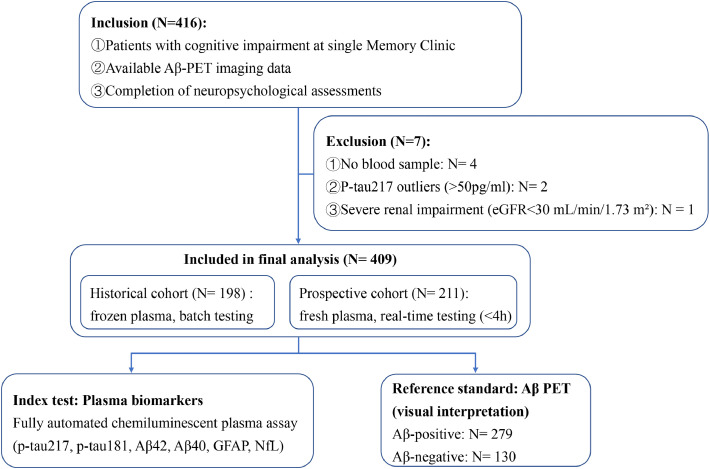
Working flowchart of participant selection in this study.

This single-center, cross-sectional, diagnostic study evaluated plasma biomarkers using amyloid-β positron emission tomography (Aβ-PET) as the reference standard (Fig. 1). Between January 2023 and June 2025, a total of 416 patients with cognitive impairment were consecutively enrolled at the Memory Clinic of Henan Provincial People's Hospital for eligibility. Inclusion criteria were as follows: (1) presence of cognitive impairment evaluated at a single-center Memory Clinic; (2) availability of Aβ-PET imaging data; and (3) completion of standardized neuropsychological assessments. Exclusion criteria included: (1) absence of a blood sample (n = 4); (2) extreme plasma p-tau217 values (>50 pg/mL), considered predefined analytical outliers (n = 2; both from frozen plasma samples); and (3) severe renal impairment, defined as an estimated glomerular filtration rate (eGFR) <30 mL/min/1.73 m² (n = 1). After exclusions, 409 participants were included in the final analysis. Blood sampling, clinical assessment, and Aβ-PET imaging were performed within the same diagnostic evaluation period, with all procedures completed within 2 weeks. Participants were categorized into two cohorts based on plasma processing and storage conditions: (1) historical cohort (n = 198), with plasma collected between January 2023 and September 2024, stored at −80 °C, and analyzed in batches; and (2) the prospective cohort (n = 211), with plasma collected between October 2024 and June 2025 and assayed on the day of collection, fresh plasma samples were collected and analyzed in real time within 4 h of blood draw. The study protocol was approved by the Institutional Ethics Committee of Henan Provincial People's Hospital (Approval No. 202,076). Written informed consent was obtained from all participants or their legally authorized representatives.

STARD flow diagram illustrating participant recruitment, plasma biomarker testing as the index test, amyloid-β positron emission tomography (Aβ-PET) as the reference standard, and inclusion in the final diagnostic accuracy analyses.

### 2.2 Diagnostic procedures

All participants underwent standardized cognitive and functional assessments, including the Clinical Dementia Rating Scale (CDR), Activities of Daily Living (ADL) scale, Huashan Auditory Verbal Learning Test (H-AVLT; delayed recall and recognition) [11], Verbal Fluency Test (VFT), Boston Naming Test (BNT), and Shape Trail Tests A and B (a validated adaptation of the Trail Making Test for Chinese to assess executive function) [12].

Dementia was diagnosed based on the following criteria: a history of cognitive decline for at least 6 months, a global CDR score ≥ 1, impaired daily functioning, and exclusion of alternative causes, such as depression, anxiety, sleep disorders, medication effects, or metabolic diseases (e.g., hypothyroidism or vitamin B12 deficiency). Mild cognitive impairment (MCI) was defined as: (1) absence of dementia (CDR < 1); (2) preserved daily functioning; and (3) fulfillment of the Jak/Bondi neuropsychological criteria—at least two test scores within the same domain or three scores across different domains falling ≥ 1.5 SD below age- and education-adjusted norms [13]. Patients were categorized into four groups based on cognitive status and Aβ-PET findings: Aβ+ dementia (n = 235), Aβ+ MCI (n = 44), Aβ– dementia (n = 77), and Aβ– MCI (n = 53).

### 2.3 Neuroimaging

All participants underwent ^18^F-AV45 PET imaging to determine cerebral Aβ status. PET scans were independently interpreted by two experienced nuclear medicine physicians blinded to all clinical information, in accordance with internationally standardized visual reading guidelines [14]. Discrepancies were resolved by a senior nuclear medicine physician.

Additionally, 95 patients underwent ^18^F AV-1451 tau PET imaging and three-dimensional T1-weighted structural magnetic resonance imaging (MRI). Tau PET quantification included standardized preprocessing, co-registration with individual T1-weighted MRI scans, and spatial normalization to the Montreal Neurological Institute (MNI) template. Partial volume correction was not applied. Standardized uptake value ratios (SUVRs) were calculated using the inferior cerebellar gray matter as the reference region. According to literature, composite cortical regions of interest were defined as follows: (1) a meta-temporal region of interest (ROI) corresponding to Braak stages I–IV and (2) a meta-neocortical ROI corresponding to Braak stages V–VI [15].

### 2.4 Plasma measurements

Blood samples were collected under standardized fasting conditions and stored at 4 °C in ethylenediaminetetraacetic acid tubes before processing. All samples were centrifuged at 1500 × g for 10 min within 2 h of collection. Historical plasma samples were frozen at –80 °C for an average of 447 ± 262 days and thawed once before batch testing. Prospective samples were processed under identical centrifugation conditions and assayed within 4 h of blood collection. Thus, the two cohorts differed in two key aspects: (1) plasma storage (frozen vs. fresh) and (2) assay mode (batch vs. real-time) (Fig. 1).

Biomarker measurements were conducted using a Vazyme® Biotech (Nanjing, China) AD plasma assay, targeting p-tau217 (M4701CB), p-tau181 (M3701CB), Aβ42 (M2701CB), Aβ40 (M1701CB), glial fibrillary acidic protein (GFAP, M5701CB), and neurofilament light chain (NfL, M6701CB). This assay was approved by the National Medical Products Administration (NMPA; registration no. 20,242,400,296). Novel antibodies for these biomarkers were generated and optimized via single B-cell cloning, screening over 50,000 clones per target. The assay consisted of antibody-coated microparticles, assay buffer, and dilution buffer and was designed as a fully automated chemiluminescent immunoassay. Plasma biomarker analysis was completed in approximately 60 min from sample loading to final results. During the development phase of the Vazyme® assay, a total of 107 freshly-collected plasma samples were used to conduct a head-to-head comparison between the ALZpath Simoa® p-tau 217 assay and the Vazyme® p-tau 217 assay. The two assays showed a strong correlation, with a correlation coefficient of 0.94.

Quality control of plasma biomarkers was rigorously implemented for each assay. Each batch included a calibration curve and low-, medium-, and high-level QC samples to monitor performance. To assess reproducibility, 10% of study samples were measured in duplicate within the same batch. Intra- and inter-assay coefficients of variation were ≤10%. The assay demonstrated a limit of detection of 0.20 pg/mL, a linear range of 0.50–50 pg/mL, and stable performance across batches.

### 2.5 Apolipoprotein E genotyping

Peripheral blood samples were collected, and DNA was extracted. Apolipoprotein E (APOE) genotype was determined using the TaqMan single-nucleotide polymorphism (SNP) method. APOE ε4 carrier status was defined as the presence of at least one ε4 allele, as APOE ε4 homozygotes are relatively uncommon in the Chinese Han population, with a reported prevalence of approximately 1%.

### 2.6 Statistical analysis

Demographic, clinical, and biomarker characteristics were described and compared across the diagnostic groups. Categorical variables were expressed as frequencies and percentages, and continuous variables were reported as medians with interquartile ranges (IQRs). Overall group differences were assessed using the nonparametric Kruskal–Wallis test, and pairwise post hoc comparisons were performed using the Mann–Whitney *U* test with Bonferroni correction.

To evaluate potential clinical and pre-analytical determinants of plasma p-tau217 levels, we performed multivariable linear regression analyses with log-transformed p-tau217 as the dependent variable. Independent variables included demographic and clinical factors (age, sex, BMI, cognitive status, Aβ-PET status, APOE ε4 carrier status, diabetes mellitus, and eGFR as well as plasma storage duration.

Receiver operating characteristic (ROC) analyses were conducted using Aβ-PET as the reference standard for individual biomarkers (p-tau217, p-tau181, p-tau217/Aβ42, NfL/p-tau217, GFAP, and Aβ42/Aβ40), as well as for p-tau217-based composite models. These analyses were performed separately in the historical and prospective cohorts. The area under the curve (AUC) values with 95% confidence intervals (estimated using bootstrap resampling), optimal cutoffs (determined using the Youden index), sensitivity, specificity, positive predictive value (PPV), negative predictive value (NPV), and overall accuracy were reported. AUCs were compared using DeLong's test. For the two cut-point strategy, bootstrap-derived cutoffs corresponding to 95% sensitivity (low threshold) and 95% specificity (high threshold) were determined for plasma p-tau217 and p-tau217/Aβ42. Participants were then stratified into three categories: negative (≤ low cutoff), positive (≥ high cutoff), and indeterminate (within the two cutoffs) [16]. The distribution of p-tau217 categories and their predictive values were reported. Sensitivity analyses were conducted across subgroups stratified by age, sex, obesity, APOE genotype, hypertension, and diabetes, as well as using quantitative Aβ-PET positivity defined by Centiloid thresholds in participants with available structural MRI data. We employed generalized additive models (GAMs) with thin plate spline smoothing functions to examine the nonlinear associations between plasma p-tau217 and regional tau-PET burden (meta-temporal and meta-neocortical ROIs). All models were adjusted for age and sex. The proportion of variance in tau-PET burden explained by p-tau217 was quantified using the adjusted R².

All statistical tests were two-sided, and statistical significance was set at P < 0.05. All analyses were conducted using the R software (version 4.1.1; R Foundation for Statistical Computing, Vienna, Austria).

## 3 Results

### 3.1 Demographic, clinical, and biomarker characteristics

This study included two cohorts: a historical cohort (n = 198) and a prospective cohort (n = 211). Based on Aβ-PET status, the historical cohort comprised 71 Aβ– and 127 Aβ+ individuals, whereas the prospective cohort comprised 59 Aβ– and 152 Aβ+ individuals. Among the 279 Aβ+ participants, 273 were diagnosed with AD dementia or AD-MCI, 3 with dementia with Lewy bodies and comorbid Aβ pathology, and 3 with cerebral amyloid angiopathy. Of the 130 Aβ– participants, 59 had vascular cognitive impairment, 36 frontotemporal lobar degeneration, 4 corticobasal degeneration, 5 progressive supranuclear palsy, 12 Parkinson's disease with cognitive impairment, 1 adult-onset leukoencephalopathy with axonal spheroids and pigmented glia, and 13 with undetermined etiology.

In the historical cohort, 81.8% of participants had dementia, and 18.2% had MCI, whereas in the prospective cohort, dementia and MCI accounted for 71.1% and 28.9% of cases, respectively. Among participants with dementia in the historical cohort, 28.3% had a CDR global score of 1, 47.0% had a CDR global score of 2, and 24.7% had a CDR global score of 3. In the prospective cohort, the corresponding proportions were 42.2%, 43.1%, and 14.7%, respectively. Demographic characteristics, comorbidities, APOE genotype, and plasma biomarker levels for the historical and prospective cohorts are summarized and compared in Table 1. In the separate analyses of the two cohorts, biomarker levels showed statistically significant and consistent differences between the Aβ+ and Aβ– groups. For instance, median plasma p-tau217 levels were elevated by approximately 3–4 fold in Aβ+ compared with Aβ– individuals (historical: 6.49 vs. 1.42 pg/mL; prospective: 6.87 vs. 1.90 pg/mL).

**Table 1:** Characteristics of demographic, clinical, and biomarkers.

Characteristics	Total			Historical			Prospective		
	Historical (n = 198)	Prospective (n = 211)	P-value	Aβ+ (n = 127)	Aβ- (n = 71)	P-value	Aβ+(n = 152)	Aβ- (n = 59)	P-value
Age, (years)	66.50 (59.00–71.75)	66.00 (59.00–74.00)	0.008	67.00 (59.00–72.00)	66.00 (59.50–70.00)	0.108	66.50 (58.00–75.25)	63.00 (59.00–72.00)	0.032
Female, n (%)	94 (47.47%)	70 (33.18%)	0.005	53 (41.73%)	41 (57.75%)	0.030	46 (30.26%)	24 (40.68%)	0.149
BMI, (kg/m²)	23.41 (21.63–25.45)	23.42 (21.48–25.27)	0.034	23.15 (21.30–25.19)	24.03 (22.00–25.94)	0.073	23.17 (21.43–25.29)	23.88 (21.54–25.18)	0.267
Education years	10.00 (8.00–12.00)	10.00 (6.50–12.00)	0.172	12.00 (7.00–12.00)	9.00 (9.00–12.00)	0.534	9.00 (6.00–12.00)	12.00 (9.00–13.00)	0.195
Subgroups			<0.001			0.006			<0.001
Dementia, n (%)	162 (81.82%)	150 (71.09%)		111 (87.40%)	51 (71.83%)		124 (81.58%)	26 (44.07%)	
MCI, n (%)	36 (18.18%)	61 (28.91%)		16 (12.60%)	20 (28.17%)		28 (18.42%)	33 (55.93%)	
Comorbidities									
Hypertension, n (%)	62 (31.31%)	56 (26.54%)	0.726	36 (28.35%)	26 (36.62%)	0.229	43 (28.29%)	13 (22.03%)	0.356
Diabetes, n (%)	26 (13.13%)	30 (14.22%)	0.055	13 (10.24%)	13 (18.31%)	0.107	19 (12.50%)	11 (18.64%)	0.251
Stroke, n (%)	34 (17.17%)	27 (12.80%)	0.094	19 (14.96%)	15 (21.13%)	0.270	17 (11.18%)	10 (16.95%)	0.261
eGFR<60 mL/ (min·1.73m²), n (%)	4 (2.84%)	4 (3.25%)	0.311	3 (3.16%)	1 (2.17%)	0.741	4 (4.26%)	0 (0.00%)	0.259
Blood biomarkers									
APOE ε4 carriers, n (%)	62 (31.79%)	93 (44.08%)	<0.001	50 (40.32%)	12 (16.90%)	<0.001	85 (55.92%)	8 (13.56%)	<0.001
p-tau217 (pg/ml)	3.94 (1.61–7.59)	5.27 (2.76–8.08)	<0.001	6.49 (3.92–8.94)	1.42 (0.87–2.18)	<0.001	6.87 (4.82–9.09)	1.90 (1.45–2.70)	<0.001
p-tau181 (pg/ml)	3.74 (1.71–5.99)	5.14 (3.05–7.18)	<0.001	5.03 (3.50–6.86)	1.67 (1.10–2.59)	<0.001	6.03 (4.66–8.27)	2.12 (1.54–3.22)	<0.001
GFAP (ln)(pg/ml)	5.09 (4.45–5.51)	5.16 (4.67–5.51)	<0.001	5.34 (4.98–5.65)	4.40 (3.89–4.86)	<0.001	5.29 (5.03–5.56)	4.44 (4.02–4.88)	<0.001
Nfl (ln)(pg/ml)	4.08 (3.66–4.55)	3.97 (3.53–4.41)	0.943	4.08 (3.75–4.50)	4.09 (3.52–4.78)	0.828	3.97 (3.60–4.40)	4.02 (3.24–4.47)	0.768
Aβ42/ Aβ40	0.06 (0.05–0.07)	0.06 (0.05–0.07)	<0.001	0.06 (0.05–0.06)	0.07 (0.06–0.07)	<0.001	0.06 (0.05–0.06)	0.07 (0.06–0.07)	<0.001
p-tau217/ Aβ42	0.58 (0.22–1.14)	0.75 (0.35–1.16)	<0.001	0.98 (0.59–1.35)	0.19 (0.11–0.29)	<0.001	0.99 (0.67–1.29)	0.24 (0.17–0.33)	<0.001
Nfl (ln)/ p-tau217	0.99 (0.57–2.22)	0.72 (0.49–1.41)	<0.001	0.65 (0.46–1.00)	2.89 (1.67–4.62)	<0.001	0.56 (0.44–0.82)	1.88 (1.50–2.48)	<0.001
tau-PET SUVR									
meta-temporal ROI	1.47 (1.26–1.97)	1.32 (1.21–1.76)	<0.001	1.66 (1.37–2.00)	1.19 (1.09–1.29)	<0.001	1.45 (1.26–1.95)	1.12 (1.08–1.17)	0.027
meta-neocortical ROI	1.29 (1.14–1.64)	1.18 (1.12–1.46)	<0.001	1.47 (1.18–1.69)	1.11 (1.04–1.19)	0.003	1.31 (1.13–1.54)	1.04 (1.03–1.12)	0.046

Abbreviations: APOE, apolipoprotein E; Aβ42, amyloid 42; NfL, neurofilament light chain; p-tau, phosphorylated tau; PET, positron emission tomography; SUVR, standardized uptake value ratio; ROI, region of interest; IQR, Quantitative variables are presented as medians.

### 3.2 Comparisons of plasma biomarkers across cognitive and Aβ status groups

**Fig. 2:**
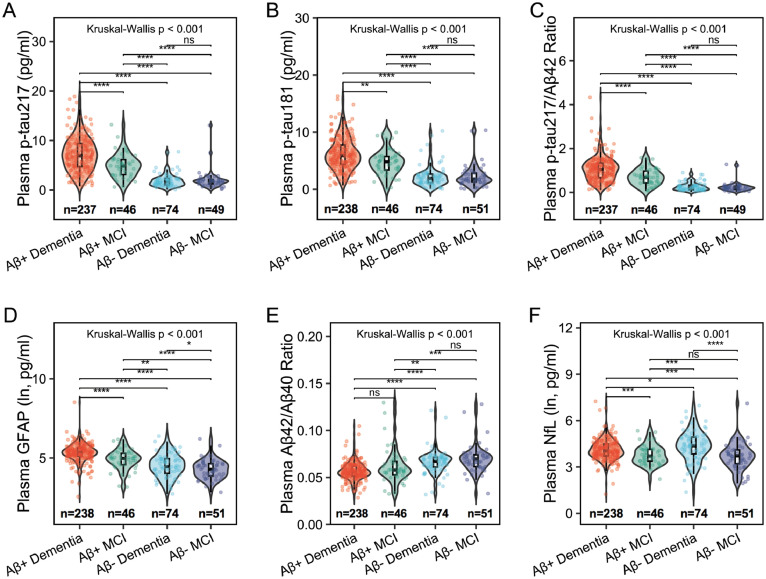
Differential expression of plasma biomarkers across subgroups.

In the pooled analysis of the two cohorts, all six plasma biomarkers significantly differed across the four diagnostic groups (Kruskal–Wallis test, P < 0.001). Plasma p-tau217, p-tau181, and the p-tau217/Aβ42 ratio were highest in the Aβ+ dementia group, intermediate in the Aβ+ MCI group, and lowest in the Aβ– groups. In contrast, the Aβ42/40 ratio was significantly lower in the Aβ+ group than in the Aβ– group. GFAP levels were significantly higher in the Aβ+ dementia group than in the Aβ+ MCI and Aβ– groups (P < 0.01). Regardless of Aβ status, plasma NfL concentrations were higher in dementia than in MCI, with the highest concentrations observed in non-AD dementia (Fig. 2).

Box-violin plots comparing p-tau217 concentrations (A), p-tau181 concentrations (B), Aβ42/Aβ40 ratio (C), p-tau217/Aβ42 ratio (D), GFAP levels (log) (E), and NfL levels (log) (F) across four diagnostic categories: Aβ+ dementia, Aβ+ MCI, Aβ-dementia, and Aβ-MCI.

### 3.3 Influence of pre-analytical and clinical variables on plasma biomarkers

To evaluate the influence of pre-analytical conditions on plasma biomarker measurements, we first compared p-tau217, p-tau181, GFAP, NfL, and Aβ42/40 ratio levels between the historical frozen-sample cohort and the prospective fresh-sample cohort within each Aβ group. Among Aβ+ participants, p-tau181 (P = 0.001) and Aβ42/40 ratio (P = 0.015) were modestly lower in the historical cohort, whereas p-tau217, GFAP, and NfL did not differ significantly between cohorts. Among Aβ− participants, p-tau217 (P = 0.001) and p-tau181 (P = 0.023) were significantly lower in the historical cohort, while GFAP, NfL, and Aβ42/40 ratio remained comparable between cohorts. Overall, these findings suggest mild reductions in selected plasma biomarker levels in historical frozen samples, particularly among Aβ− participants (Table S1).

We next evaluated potential clinical and pre-analytical determinants of plasma p-tau217 concentrations using multivariable linear regression analyses, adjusting for age, sex, BMI, cognitive status, Aβ status, APOE ε4 carrier status, diabetes mellitus, eGFR, and plasma storage duration. After multivariable adjustment, Aβ positivity remained the strongest predictor of higher plasma p-tau217 concentrations (β = 1.194, P < 0.001). Longer storage duration was independently associated with lower plasma p-tau217 levels (β = −0.012 per month, P < 0.001), corresponding to an approximate decline of 1.2% per month or ∼14% per year. APOE ε4 carrier status was also independently associated with higher plasma p-tau217 concentrations (β = 0.194, P = 0.021). In contrast, age, sex, BMI, diabetes status, eGFR, and cognitive status were not independently associated with plasma p-tau217 concentrations after multivariable adjustment (Table S2).

### 3.4 Diagnostic performance of plasma biomarkers for Aβ pathology

**Fig. 3:**
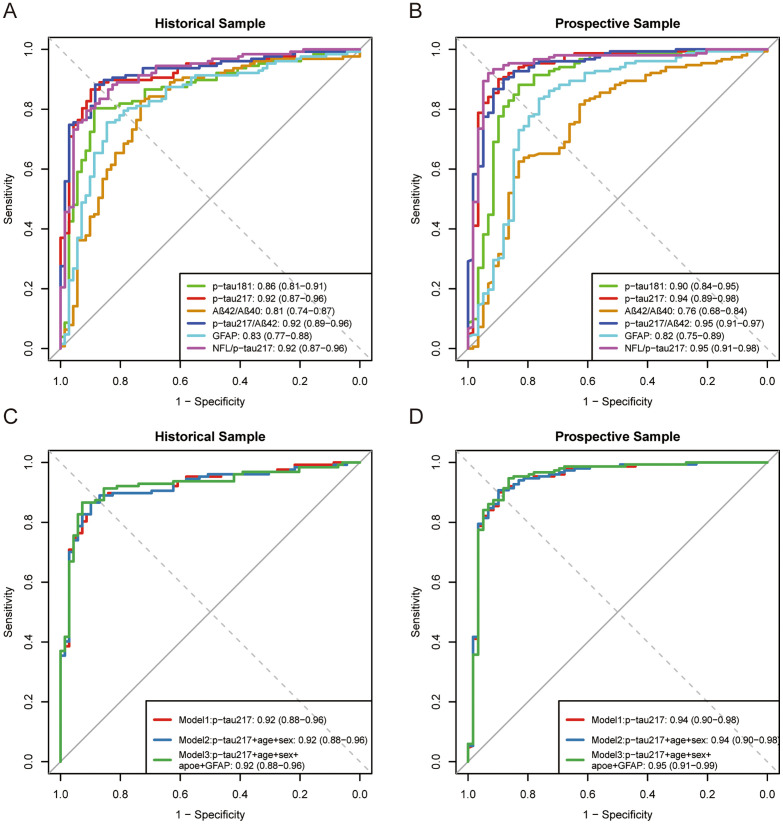
Receiver operating characteristic analysis of plasma biomarkers for Aβ status.

In the historical cohort, p-tau217, p-tau217/Aβ42 ratio, and NfL /p-tau217 ratio showed the highest accuracy for discriminating Aβ pathology, with all AUCs of 0.92. The AUCs for p-tau181, GFAP, and the Aβ42/40 ratio were 0.86, 0.83, and 0.81, respectively. In the prospective cohort, the p-tau217, p-tau217/Aβ42 ratio, and NfL/p-tau217 ratio performed superiorly, with respective AUCs of 0.94, 0.95, and 0.95. The p-tau181 ratio achieved an AUC of 0.90, the GFAP 0.82, and the Aβ42/40 ratio 0.76 (Fig. 3).

Receiver Operating Characteristic (ROC) curves evaluating six plasma biomarkers for distinguishing amyloid-β positive (Aβ+) from amyloid-β negative (Aβ-) individuals in the historical cohort (A, C) and prospective cohort (B, D).

Combining p-tau217 with other biomarkers did not significantly improve diagnostic performance compared to using p-tau217 alone. In the historical cohorts, Model 1 (p-tau217), Model 2 (p-tau217 + age + sex), and Model 3 (p-tau217 + age + sex + GFAP + APOE) all achieved an AUC of 0.92, with no significant pairwise differences (DeLong test, all P > 0.05). In the prospective cohorts, Models 1 (p-tau217), 2 (p-tau217 + age + sex), and 3 (p-tau217 + age + sex + GFAP + APOE) each achieved an AUC of 0.94, 0.94, and 0.95 (Fig. 3). However, Model 3 produced the highest Youden index at the optimal cutoff (Table S3).

### 3.5 Classification of Aβ status using a two-cut-point strategy

Two cut-points corresponding to 95% sensitivity and specificity were applied to both p-tau217 and the p-tau217/Aβ42 ratio. In the historical cohort, the cut-points for p-tau217 were 1.54 (low) and 3.94 (high), yielding a PPV of 0.97 and an NPV of 0.86. For the p-tau217/Aβ42 ratio, the cut-points were 0.17 (low) and 0.57 (high), yielding a PPV of 0.97 and an NPV of 0.86. In the prospective cohort, the p-tau217 cut-points were 2.77 (low) and 4.66 (high), yielding a PPV of 0.99 and an NPV of 0.87. For the p-tau217/Aβ42 ratio, the cut-points were 0.36 (low) and 0.81 (high), yielding a PPV of 0.99 and an NPV of 0.87.

**Fig. 4:**
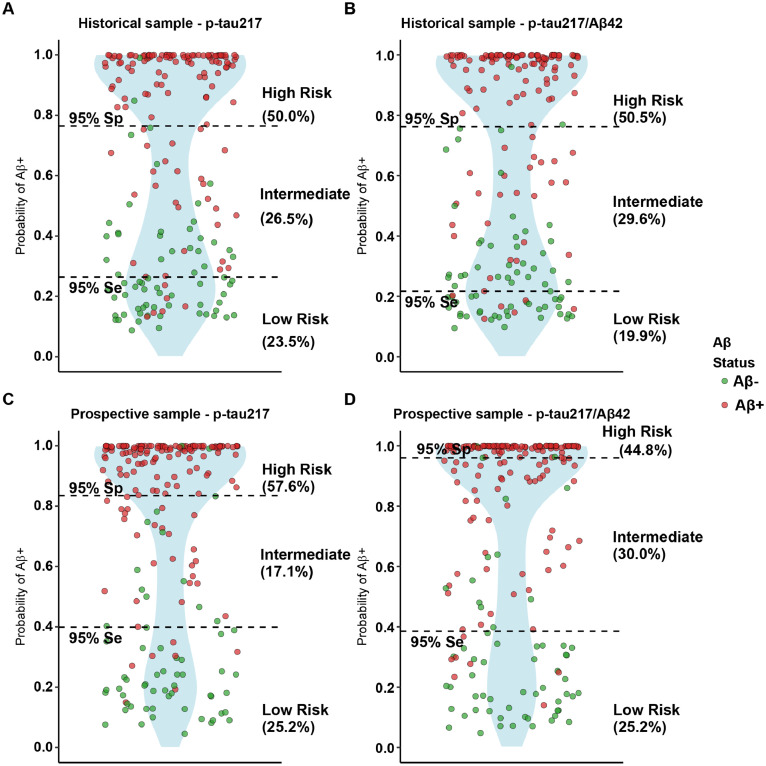
Risk stratification by two-cut-point approach of plasma p-tau217 and p-tau217/Aβ42 ratio.

The indeterminate zone included 26.5% of cases in the historical cohort and 17.1% in the prospective cohort for p-tau217, and 29.6% and 30.0% of cases, respectively, for the p-tau217/Aβ42 ratio (Figs. 4, S1).

Distribution of plasma p-tau217 levels and associated amyloid positivity probability in the historical (A) and prospective (C) cohorts. Corresponding probability curves for amyloid positivity based on p-tau217/Aβ42 levels within each risk category for the historical (B) and prospective (D) cohorts.

### 3.6 Stratified analysis of diagnostic performance for p-tau217 and p-tau217/Aβ42 ratio

Sensitivity analyses were performed to evaluate the robustness of plasma p-tau217 and the p-tau217/Aβ42 ratio across clinically relevant subgroups stratified by sex, age (≤65 vs. >65 years), APOE ε4 carrier status, obesity (BMI ≥25 kg/m²), hypertension, and diabetes mellitus. Overall, diagnostic performance remained consistently high across subgroups, although slightly lower AUCs with wider confidence intervals were observed among APOE ε4 carriers (AUC 0.89, 95% CI: 0.78–0.98) and participants with diabetes mellitus (AUC 0.90, 95% CI: 0.80–0.97) (Fig. S2).

### 3.7 Association between p-tau217 and Tau-PET burden

Linear correlation analysis revealed a significant association between p-tau217 and tau-PET SUVR uptake in both meta-temporal ROI and neocortical ROI (r = 0.58, 0.51, both P < 0.001). This correlation is absent in Aβ– samples. In generalized additive models, plasma p-tau217 demonstrated significant nonlinear associations with tau-PET burden (both P < 0.001). The adjusted R^2^ was 0.43 for the meta-temporal ROI and 0.38 for the meta-neocortical ROI (Fig. S3).

## 4 Discussion

In this study, we validated the diagnostic performance of the novel Vazyme® plasma biomarker assays for AD in distinguishing Aβ pathology among memory clinic patients under two pre-analytical conditions. The findings demonstrated that both batched testing of frozen plasma samples and real-time testing of fresh samples exhibited excellent discriminative performance for p-tau217, p-tau217/Aβ42 ratio, and NfL/p-tau217 ratio, with AUC values ranging from 0.92 to 0.95. The single p-tau217 model demonstrated a diagnostic performance comparable to that of integrated biomarker models. By applying a dual-threshold strategy, the indeterminate zone was restricted to <30%, while yielding better positive and negative predictive values. These findings confirm the robustness of this novel plasma assay under two pre-analytical conditions, with superior performance in fresh plasma, thereby supporting its integration into real-world diagnostic workflows for AD.

Plasma p-tau217 has emerged as one of the most promising blood-based biomarkers for AD because of its strong association with cerebral amyloid and tau pathology. Although plasma p-tau217 has shown robust diagnostic performance across multiple international cohorts, validation data in Chinese clinical populations remain relatively limited. In addition, most currently available assays rely on a limited number of p-tau217 antibody clones, highlighting the importance of validating newly developed antibody combinations. In the present study, we evaluated a newly developed automated chemiluminescent plasma biomarker assay incorporating six AD-related biomarkers in a real-world Chinese memory clinic cohort and provided an initial clinical validation of this assay under different pre-analytical conditions. The assay is implemented on a routine automated chemiluminescent platform that may be more readily integrated into existing laboratory workflows in primary and regional healthcare settings.

This study examined the diagnostic performance in two scenarios: frozen biobanked plasma compatible with retrospective analysis and fresh plasma applicable to real-world clinical use. In the prospective cohort, p-tau217, p-tau217/Aβ42, and NfL/p-tau217 ratios achieved higher discriminative accuracy than in the historical cohort (AUC 0.94–0.95 vs. 0.92). This finding indicates that the timely testing of plasma samples is of greater importance than batch processing. At optimal cutoffs, p-tau217 or its ratio demonstrated sensitivity and specificity exceeding 90% in the prospective cohort, which meets the Alzheimer's Association's recommended criteria for confirmatory blood biomarker testing [17]. Dual-threshold analyses further yielded an excellent PPV of 0.97–0.99, NPV of 0.86–0.87, and indeterminate zones < 30%, similar to results reported abroad [[18], [19], [20]]. The relatively lower NPVs likely reflect the high prevalence of Aβ pathology (70%) in this cohort, consistent with the theorized inverse relationship between NPV and disease prevalence.

Previous studies have reported the effectiveness of the NfL/p-tau217 ratio in distinguishing AD from non-AD dementias, and our findings are consistent with these reports [21]. This is likely related to the spectrum of non-AD dementias in our sample—primarily vascular dementia (VaD, 59/130) and other neurodegenerative dementias such as FTD, CBD, PSP, and PDD (45/130)—which tend to exhibit markedly elevated plasma NfL levels without corresponding increases in p-tau217. By combining these biomarkers into a ratio, the differential signals are amplified, enhancing discrimination between AD and non-AD dementias. Additionally, the relative stability and low degradation susceptibility of NfL may further contribute to the robustness of this composite marker [22]. Nevertheless, further validation across a broader spectrum of dementia etiologies is warranted to confirm its generalizability and clinical interpretability.

In stratified analyses, the diagnostic performance of plasma p-tau217 was slightly reduced in APOE ε4 carriers and patients with diabetes. A multicenter Lumipulse study in symptomatic cognitively impaired individuals reported a similar minor decrease in Aβ discrimination (AUC 0.95→ 0.93) [23]. This reduction may partly reflect the limited sample sizes of these subgroups, but true biological modulation may also contribute. Mechanistically, in APOE ε4 carriers, tau phosphorylation may be elevated even in Aβ– individuals via GSK-3β activation, inflammation, and microglial activity, reducing the difference between Aβ+ and Aβ– groups [24]. In patients with diabetes, insulin resistance, metabolic dysregulation, inflammation, and blood–brain barrier changes may increase tau phosphorylation and plasma p-tau217 release [25]. These non-specific elevations can weaken its discriminative power for Aβ pathology.

In this study, shifts in cutoff values and discriminative performance were observed between the two cohorts. Specifically, the thresholds for p-tau217 and the p-tau217/Aβ42 ratio were higher in the prospective cohort, whereas the threshold for the NfL/p-tau217 ratio was lower. These differences may reflect the combined influence of freeze–thaw effects, storage duration, and differences in dementia severity between the two cohorts [26]. After multivariable adjustment, storage time was significantly negatively associated with log-transformed p-tau217 (β = −0.012 per month), corresponding to a cumulative decline of ∼14% per year. It is worth noting that, in this study, the historical cohort consisted of frozen samples, which necessarily underwent a single thaw–freeze cycle. Therefore, storage time and the thaw–freeze process are completely collinear, and the estimated effect of storage time reflects the combined impact of storage duration and a single thaw–freeze cycle.

The cohort in this study was derived from a single tertiary hospital memory clinic, in which nearly 70% of participants were Aβ-positive, representing a highly enriched population. Using data from our prospective cohort, we identified an optimal p-tau217 threshold of 3.51 pg/mL. However, disease prevalence must be explicitly considered when interpreting plasma p-tau217 results and applying them across different clinical or research settings [27]. We calculated the corresponding PPV and NPV for this threshold across populations with varying Aβ prevalence (Table S4). In low-prevalence community screening settings, NPV is very high, whereas PPV is substantially reduced. Therefore, when applying plasma p-tau217 for community-based screening, threshold values should be adjusted to achieve a desired target PPV.

The strengths of this study include the use of Aβ-PET as the reference standard, validation across two pre-analytical conditions, and the establishment of preliminary cutoff values. In addition to predicting Aβ pathology, this study also demonstrated a strong correlation between p-tau217 and tau-PET burden, with a determination coefficient consistent with prior studies [28]. Several limitations should be acknowledged. First, participants were recruited from a single tertiary memory clinic, resulting in a dementia-enriched cohort with a relatively limited proportion of MCI and Aβ− individuals. Therefore, the diagnostic performance and cutoff thresholds observed in this study should be considered preliminary and may not be directly generalizable to broader clinical populations. Second, while we additionally evaluated several clinical and pre-analytical variables, many potentially relevant pre-analytical factors and comorbidity-related influences on plasma biomarkers were not comprehensively assessed in the current study. Third, visual Aβ-PET assessment was used as the primary reference standard because structural MRI data required for quantitative Centiloid analyses were unavailable in approximately half of the participants. Further multicenter validation studies and head-to-head comparisons with established plasma p-tau217 platforms are still required.

## 5 Conclusions

In conclusion, this study provides an initial clinical validation of a fully automated chemiluminescent plasma biomarker assay for detecting Aβ pathology in a Chinese memory clinic cohort under different pre-analytical conditions. Plasma p-tau217 demonstrated robust and consistent diagnostic performance across both historical frozen samples and freshly analyzed real-time samples. These findings support the potential utility of automated plasma biomarker platforms in routine memory clinic practice, while further multicenter studies and head-to-head comparisons with established assays remain necessary before broader clinical implementation.

## Ethics approval and consent to participate

The study protocol was approved by the Institutional Ethics Committee of Henan Provincial People's Hospital (Approval No. 202,076).

## Consent for publication

Written informed consent was obtained from all participants or their legally authorized representatives.

## Availability of data and materials

The data used and analyzed during this study are available from the corresponding author on reasonable request.

## Funding

This study was supported by the Henan Province Key R&D Program (241111313500), the National Natural Science Foundation of China (82201471), and the Henan Provincial International Science and Technology Cooperation Program (252102521060).

## CRediT authorship contribution statement

**Shuai Chen:** Writing – review & editing, Writing – original draft, Supervision, Software, Conceptualization. **Feng-Yu Wang:** Writing – original draft, Methodology, Data curation. **Rong Li:** Writing – original draft, Project administration, Data curation. **Chang Fu:** Software, Project administration, Methodology. **Jing-Yu Shao:** Visualization, Validation, Data curation. **Yu Shen:** Resources, Project administration, Methodology, Data curation. **Kai Ma:** Writing – original draft, Software, Methodology, Data curation. **Xiao-Di Hao:** Writing – original draft, Funding acquisition. **Lin Cao:** Writing – review & editing, Methodology, Data curation. **Jun-Ling Xu:** Writing – review & editing, Supervision, Data curation, Conceptualization. **Jie-Wen Zhang:** Writing – review & editing, Supervision, Resources, Funding acquisition, Conceptualization.

## Declaration of competing interest

The authors declared no potential conflicts of interest concerning the research, authorship, and/or publication of this article.

## Appendix Supplementary materials

Image, application 1
