# Supplementary material for: Diagnostic performance of an automated plasma p-tau217 chemiluminescent assay for detecting Aβ pathology in a Chinese memory clinic cohort
Source: J Prev Alzheimers Dis. 2026 Jun 5;13(7):100613. doi: 10.1016/j.tjpad.2026.100613 (PMC13266171; doi:10.1016/j.tjpad.2026.100613)
Supplement: Supplementary file 1 [file mmc1.zip › Figure S1.pdf]

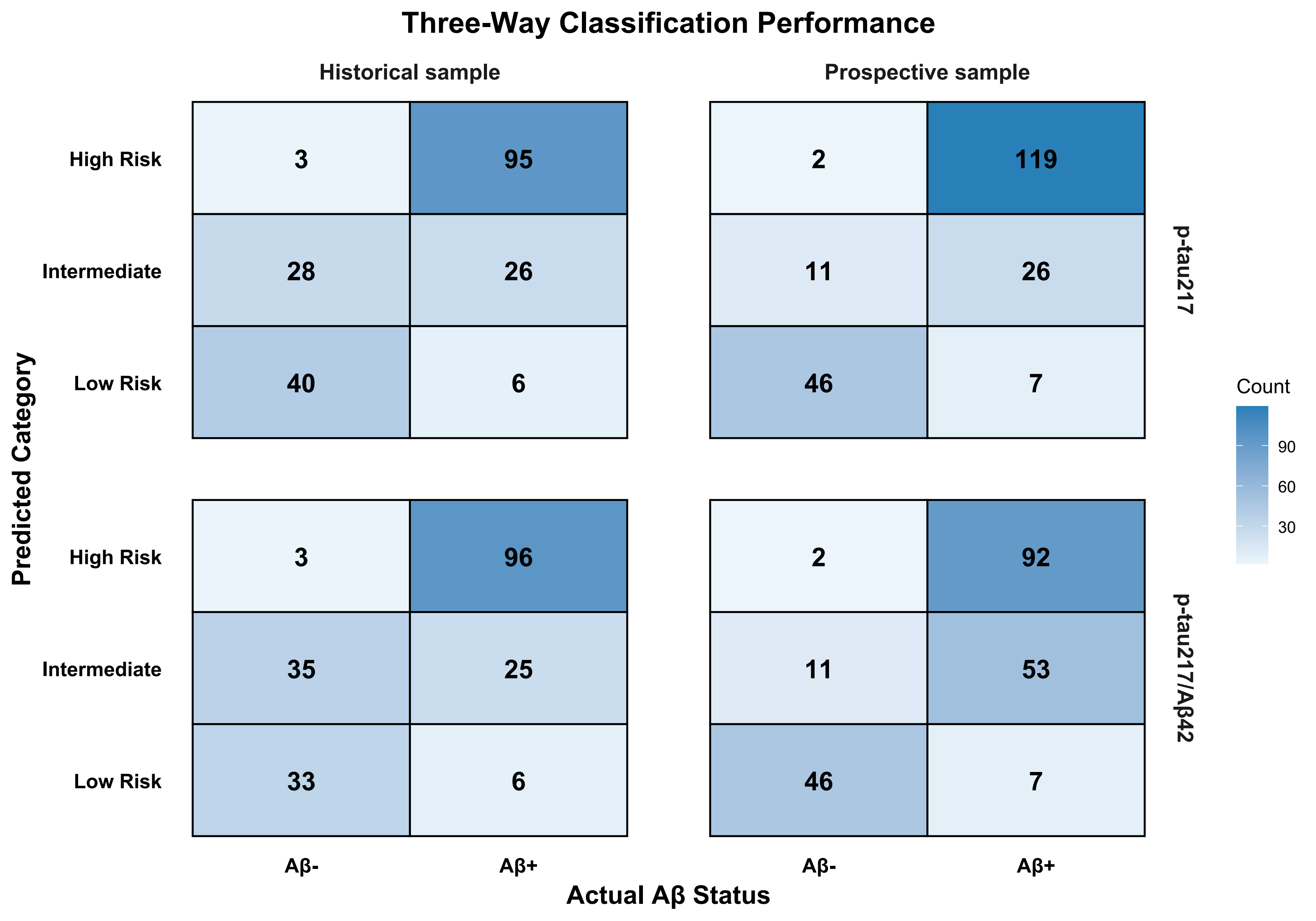

Three-Way Classification Performance
Historical sample
Prospective sample
Predicted Category
High Risk
Intermediate
Low Risk
3
95
28
26
40
6
2
119
11
26
46
7
p-tau217
High Risk
Intermediate
Low Risk
3
96
35
25
33
6
2
92
11
53
46
7
p-tau217/Aβ42
Count
90
60
30
Aβ-
Aβ+
Aβ-
Aβ+
Actual Aβ Status
