# Supplementary material for: Diagnostic performance of an automated plasma p-tau217 chemiluminescent assay for detecting Aβ pathology in a Chinese memory clinic cohort
Source: J Prev Alzheimers Dis. 2026 Jun 5;13(7):100613. doi: 10.1016/j.tjpad.2026.100613 (PMC13266171; doi:10.1016/j.tjpad.2026.100613)
Supplement: Supplementary file 1 [file mmc1.zip › TABLE S1.docx]

­ Table S1. Baseline characteristics of participants across different cohorts stratified by Aβ status.

| Characteristics | Aβ+ | | | Aβ- | | |
| --- | --- | --- | --- | --- | --- | --- |
|  | HISTORICAL(n=127) | PROSPERECTIVE(n=152) | P-value | HISTORICAL(n=71) | PROSPERECTIVE(n=59) | P-value |
| Age, (years, IQR) | 67.00 (59.00-72.00) | 66.50 (58.00-75.25) | 0.703 | 66.00 (59.50-70.00) | 63.00 (59.00-72.00) | 0.989 |
| Female, n (%) | 53 (41.73%) | 46 (30.26%) | 0.062 | 41 (57.75%) | 24 (40.68%) | 0.078 |
| BMI, (kg/m², IQR) | 23.15 (21.30-25.19) | 23.17 (21.43-25.29) | 0.927 | 24.03 (22.00-25.94) | 23.88 (21.54-25.18) | 0.509 |
| EDUCATION, (YEARS, IQR) | 12.00 (7.00-12.00) | 9.00 (6.00-12.00) | 0.786 | 9.00 (9.00-12.00) | 12.00 (9.00-13.00) | 0.499 |
| Subgroups |  | | 0.244 |  | | **0.002** |
| Dementia, n (%) | 111 (87.40%) | 124 (81.58%) |  | 51 (71.83%) | 26 (44.07%) |  |
| MCI, n (%) | 16 (12.60%) | 28 (18.42%) |  | 20 (28.17%) | 33 (55.93%) |  |
| Comorbidities | | | | | | |
| Hypertension, n (%) | 36 (28.35%) | 43 (28.29%) | 1.000 | 26 (36.62%) | 13 (22.03%) | 0.106 |
| Diabetes, n (%) | 13 (10.24%) | 19 (12.50%) | 0.687 | 13 (18.31%) | 11 (18.64%) | 1.000 |
| Stroke, n (%) | 19 (14.96%) | 17 (11.18%) | 0.449 | 15 (21.13%) | 10 (16.95%) | 0.705 |
| eGFR<60 mL/ (min·1.73m²), n (%) | 3 (3.16%) | 4 (4.26%) | 0.990 | 1 (2.17%) | 0 (0.00%) | 1.000 |
| Blood biomarkers | | | | | | |
| APOE ε4 carriers, n (%) | 50 (40.32%) | 85 (55.92%) | 0.890 | 12 (16.90%) | 8 (13.56%) | 0.865 |
| p-tau217 (pg/ml, IQR) | 6.49 (3.92-8.94) | 6.87 (4.82-9.09) | 0.196 | 1.42 (0.87-2.18) | 1.90 (1.45-2.70) | **0.001** |
| p-tau181 (pg/ml, IQR) | 5.03 (3.50-6.86) | 6.03 (4.66-8.27) | **0.001** | 1.67 (1.10-2.59) | 2.12 (1.54-3.22) | **0.023** |
| GFAP (ln)(pg/ml, IQR) | 5.34 (4.98-5.65) | 5.29 (5.03-5.56) | 0.783 | 4.40 (3.89-4.86) | 4.44 (4.02-4.88) | 0.425 |
| Nfl (ln)(pg/ml, IQR) | 4.08 (3.75-4.50) | 3.97 (3.60-4.40) | 0.110 | 4.09 (3.52-4.78) | 4.02 (3.24-4.47) | 0.367 |
| Aβ42/ Aβ40 | 0.06 (0.05-0.06) | 0.06 (0.05-0.06) | **0.015** | 0.07 (0.06-0.07) | 0.07 (0.06-0.07) | 0.567 |
| p-tau217/ Aβ42 | 0.98 (0.59-1.35) | 0.99 (0.67-1.29) | 0.978 | 0.19 (0.11-0.29) | 0.24 (0.17-0.33) | **0.002** |
| Nfl (ln)/ p-tau217 | 0.65 (0.46-1.00) | 0.56 (0.44-0.82) | **0.031** | 2.89 (1.67-4.62) | 1.88 (1.50-2.48) | **0.001** |
| tau-PET SUVR | | | | | | |
| meta-temporal ROI | 1.66 (1.37-2.00) | 1.45 (1.26-1.95) | 0.248 | 1.19 (1.09-1.29) | 1.12 (1.08-1.17) | 0.387 |
| meta-neocortical ROI | 1.47 (1.18-1.69) | 1.31 (1.13-1.54) | 0.217 | 1.11 (1.04-1.19) | 1.04 (1.03-1.12) | 0.387 |

Abbreviations: APOE, apolipoprotein E; Aβ42, amyloid 42; NfL, neurofilament light chain; p-tau, phosphorylated tau; PET, positron emission tomography; SUVR, standardized uptake value ratio; ROI, region of interest; IQR, Quantitative variables are presented as medians.
