# Supplementary material for: Diagnostic performance of an automated plasma p-tau217 chemiluminescent assay for detecting Aβ pathology in a Chinese memory clinic cohort
Source: J Prev Alzheimers Dis. 2026 Jun 5;13(7):100613. doi: 10.1016/j.tjpad.2026.100613 (PMC13266171; doi:10.1016/j.tjpad.2026.100613)
Supplement: Supplementary file 1 [file mmc1.zip › Table S2.docx]

**Table S2. Multivariable linear regression analysis of factors associated with plasma p-tau217.**

| Variable | Estimate (β) | 95% CI | P value |
| --- | --- | --- | --- |
| Intercept | 1.868 | 0.488 to 3.247 | 0.009 |
| Age | −0.008 | −0.018 to 0.002 | 0.126 |
| Sex | 0.114 | −0.045 to 0.274 | 0.162 |
| Aβ status | 1.194 | 0.998 to 1.390 | <0.000001 |
| Cognitive status | 0.148 | −0.044 to 0.341 | 0.133 |
| Storage time (mo) | −0.012 | −0.026 to −0.008 | <0.001 |
| BMI | −0.016 | −0.044 to 0.013 | 0.281 |
| eGFR | −0.006 | −0.013 to 0.001 | 0.081 |
| Diabetes mellitus | −0.120 | −0.337 to 0.098 | 0.283 |
| APOE ε4 carrier | 0.194 | 0.030 to 0.357 | 0.021 |
