# Supplementary material for: Diagnostic performance of an automated plasma p-tau217 chemiluminescent assay for detecting Aβ pathology in a Chinese memory clinic cohort
Source: J Prev Alzheimers Dis. 2026 Jun 5;13(7):100613. doi: 10.1016/j.tjpad.2026.100613 (PMC13266171; doi:10.1016/j.tjpad.2026.100613)
Supplement: Supplementary file 1 [file mmc1.zip › TABLE S3.docx]

**Table S3. Diagnostic value of plasma biomarkers and biomarker combinations in different models.**

|  | Historical Batch | | | | | | Prospective Batch | | | | | |
| --- | --- | --- | --- | --- | --- | --- | --- | --- | --- | --- | --- | --- |
|  | **Optimal**  **threshold** | **Accuracy** | **sensitivity** | **specificity** | **PPV** | **NPV** | **Optimal**  **threshold** | **Accuracy** | **sensitivity** | **specificity** | **PPV** | **NPV** |
| p-tau181 | 3.12 | 0.83 | 0.80 | 0.89 | 0.93 | 0.72 | 3.67 | 0.87 | 0.88 | 0.83 | 0.93 | 0.73 |
| p-tau217 | 2.70 | 0.88 | 0.87 | 0.90 | 0.94 | 0.78 | 3.51 | 0.90 | 0.90 | 0.90 | 0.96 | 0.79 |
| Aβ42/Aβ40 | 0.06 | 0.79 | 0.83 | 0.73 | 0.85 | 0.70 | 0.06 | 0.68 | 0.63 | 0.83 | 0.90 | 0.46 |
| p-tau217/Aβ42 | 0.38 | 0.88 | 0.88 | 0.88 | 0.93 | 0.80 | 0.49 | 0.88 | 0.87 | 0.92 | 0.96 | 0.73 |
| GFAP | 143.70 | 0.79 | 0.76 | 0.85 | 0.90 | 0.66 | 132.67 | 0.82 | 0.84 | 0.76 | 0.90 | 0.64 |
| NfL (ln)/p-tau217 | 1.08 | 0.84 | 0.80 | 0.91 | 0.94 | 0.71 | 1.16 | 0.92 | 0.92 | 0.93 | 0.97 | 0.82 |
| p‑tau217 + age + sex | 0.51 | 0.88 | 0.87 | 0.90 | 0.94 | 0.78 | 0.59 | 0.90 | 0.91 | 0.90 | 0.96 | 0.79 |
| p‑tau217 + age + sex +GFAP + APOE | 0.57 | 0.89 | 0.87 | 0.93 | 0.96 | 0.79 | 0.53 | 0.92 | 0.95 | 0.86 | 0.95 | 0.86 |

Abbreviations: APOE, apolipoprotein E; Aβ42, amyloid 42; GFAP, glial fibrillary acidic protein; NFL, neurofilament light chain; p-Tau, phosphorylated tau; PPV, positive predictive value; NPV, negative predictive value.
