# Supplementary material for: Diagnostic performance of an automated plasma p-tau217 chemiluminescent assay for detecting Aβ pathology in a Chinese memory clinic cohort
Source: J Prev Alzheimers Dis. 2026 Jun 5;13(7):100613. doi: 10.1016/j.tjpad.2026.100613 (PMC13266171; doi:10.1016/j.tjpad.2026.100613)
Supplement: Supplementary file 1 [file mmc1.zip › Table S4.docx]

**Table S4. PPV and NPV of p-tau217 across different Aβ prevalence**

| Assumed prevalences of Aβ pathology | PPV | NPV |
| --- | --- | --- |
| Cognitive impaired |  | |
| 0.7 (tertiary memory clinic) | 0.954 | 0.795 |
| 0.5 (primary care) | 0.899 | 0.900 |
| Cognitive unimpaired (community screening) |  | |
| 0.38 (age 85) | 0.844 | 0.937 |
| 0.28 (age 75) | 0.775 | 0.959 |
| 0.19 (age 65) | 0.675 | 0.975 |
