# Supplementary figures and images for: Diagnostic performance of an automated plasma p-tau217 chemiluminescent assay for detecting Aβ pathology in a Chinese memory clinic cohort
Source: J Prev Alzheimers Dis. 2026 Jun 5;13(7):100613. doi: 10.1016/j.tjpad.2026.100613 (PMC13266171; doi:10.1016/j.tjpad.2026.100613)

A

Plasma p-tau217

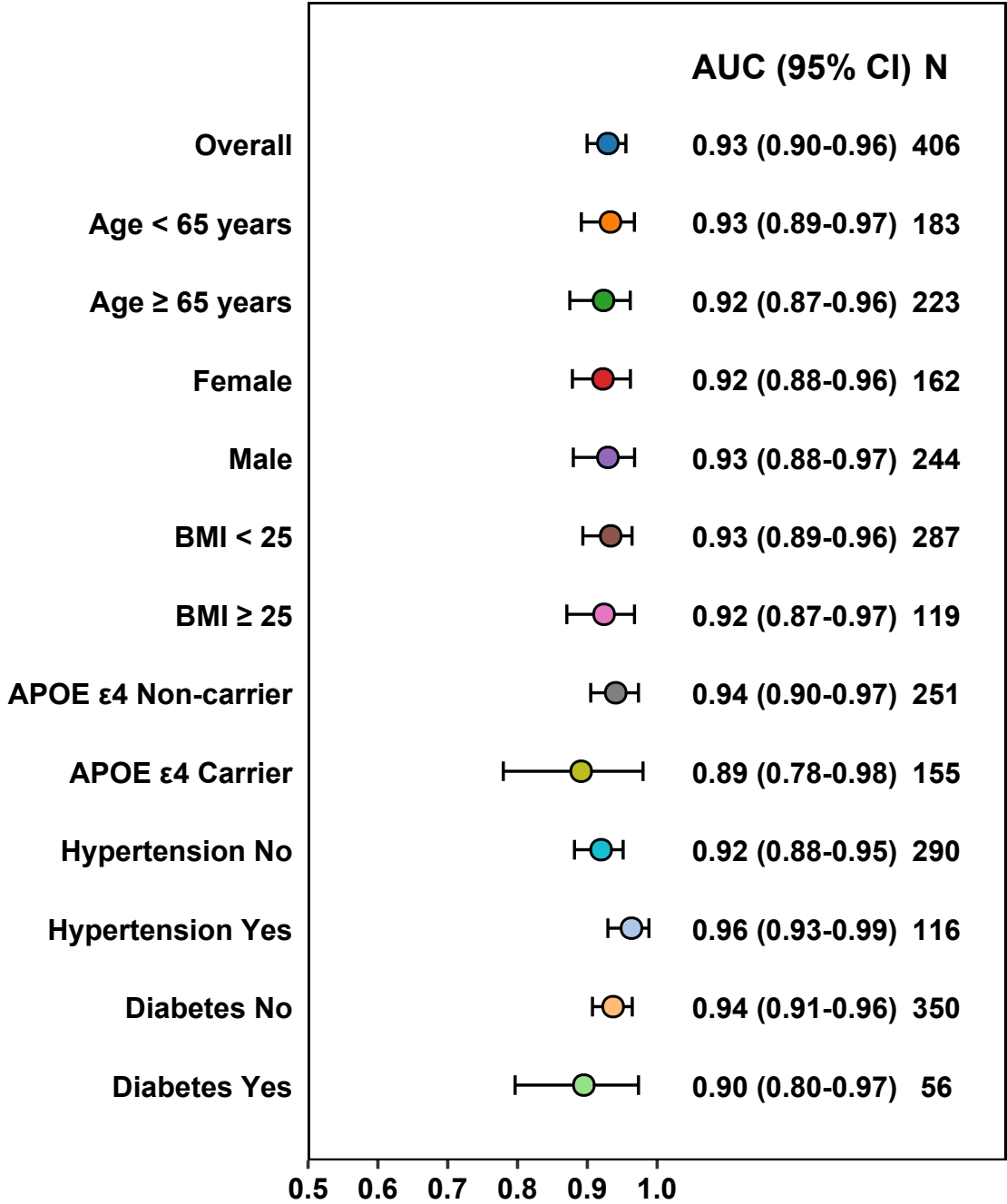

B

Plasma p-tau217/Aβ42

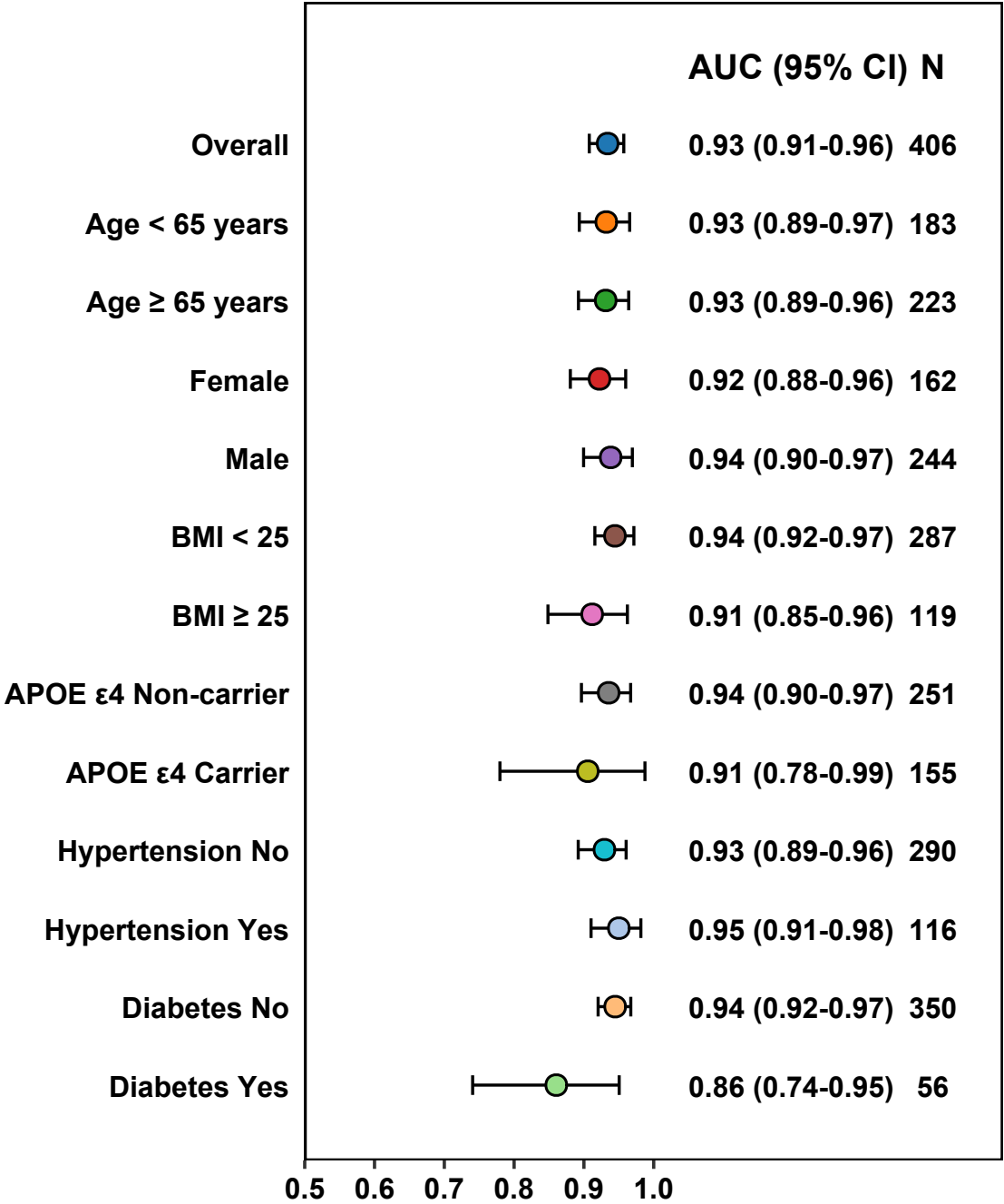

Supplement: Supplementary file 1 [file mmc1.zip › Figure S2.pdf]

**A****Tau-PET Temporal ROI vs p-tau217**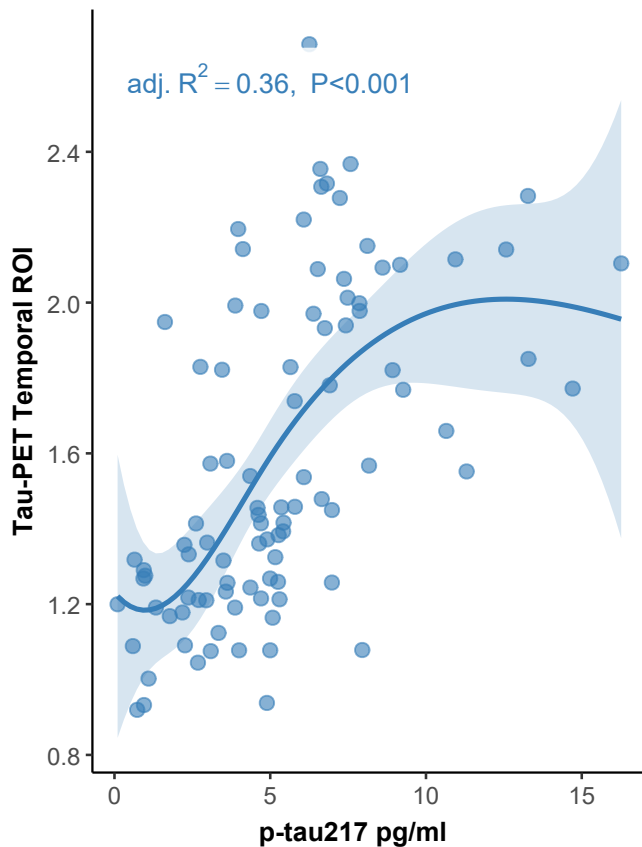**B****Tau-PET Neocortical ROI vs p-tau217**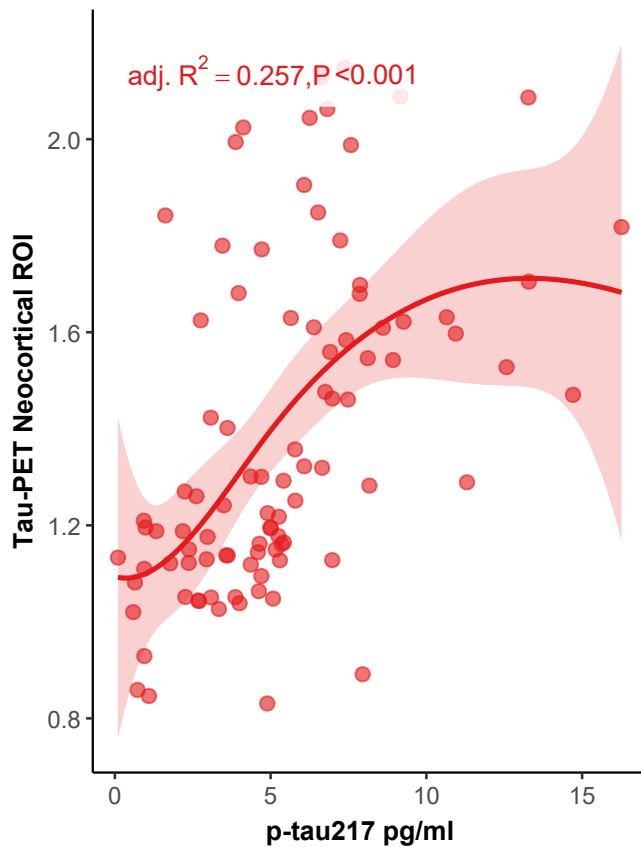

Supplement: Supplementary file 1 [file mmc1.zip › Figure S3.pdf]
